# A Thermo-Responsive Polymer Micelle with a Liquid Crystalline Core

**DOI:** 10.3390/polym15030770

**Published:** 2023-02-02

**Authors:** Yoko Mizoue, Rintaro Takahashi, Kazuo Sakurai, Shin-ichi Yusa

**Affiliations:** 1 Department of Applied Chemistry, Graduate School of Engineering, University of Hyogo, 2167 Shosha, Himeji 671-2280, Hyogo, Japan; 2 Department of Energy Engineering, Graduate School of Engineering, Nagoya University, Furo-cho, Chikusa-ku, Nagoya 464-8603, Aichi, Japan; 3 Department of Chemistry and Biochemistry, University of Kitakyushu, 1-1 Hibikino, Kitakyushu 808-0135, Fukuoka, Japan

**Keywords:** cholesterol, lower critical solution temperature (LCST), liquid crystal, amphiphilic copolymer, reversible addition–fragmentation chain transfer (RAFT)

## Abstract

An amphiphilic diblock copolymer (PChM-PNIPAM), composed of poly(cholesteryl 6-methacryloyloxy hexanoate) (PChM) and poly(*N*-isopropyl acrylamide) (PNIPAM) blocks, was prepared via reversible addition–fragmentation chain transfer radical polymerization. The PChM and PNIPAM blocks exhibited liquid crystalline behavior and a lower critical solution temperature (LCST), respectively. PChM-PNIPAM formed water-soluble polymer micelles in water below the LCST because of hydrophobic interactions of the PChM blocks. The PChM and PNIPAM blocks formed the core and hydrophilic shell of the micelles, respectively. With increasing temperature, the molecular motion of the pendant cholesteryl groups increased, and a liquid crystalline phase transition occurred from an amorphous state in the core. With further increases in temperature, the PNIPAM block in the shell exhibited the LCST and dehydrated. Hydrophobic interactions of the PNIPAM shells resulted in inter-micellar aggregation above the LCST.

## 1. Introduction

Cholesterol is a lipid sterol and a component of the eukaryotic cell membrane that plays a vital role in regulating the fluidity and permeability of the membrane [1,2]. Synthetic polymers containing cholesterol as a biological substance have attracted attention in biomedical fields, such as bioimaging and drug delivery systems (DDS) [3]. Additionally, cholesterol derivatives produce cholesteric liquid crystals [4]. Cholesteric liquid crystals have a spiral structure, in which the layers of oriented mesogens are twisted [5]. Generally, liquid crystals are classified into nematic, smectic, and cholesteric phases according to their orientation [6]. Additionally, liquid crystals are classified into thermotropic and lyotropic liquid crystals [7]. Thermotropic liquid crystals show a liquid crystalline phase induced by temperature, and lyotropic liquid crystals show a liquid crystalline phase in solution. Liquid crystals have attracted interest in biology and materials chemistry as dynamic functional soft materials [8].

Amphiphilic block copolymers have applications in water-based paints, coatings, cosmetics, foods, personal care products, DDS, and water treatment [9,10,11]. Generally, amphipathic diblock copolymers form interpolymer aggregates through the association of the hydrophobic blocks in water [12]. The shape of the polymer aggregate depends on the molecular weight balance between the hydrophilic and hydrophobic blocks, the chemical structures of each block, and the degree of polymerization (DP) [13,14,15,16]. The shape of polymer aggregates in water changes to spherical micelles, worm-like micelles, and vesicles with increasing DP of the hydrophobic block or decreasing DP of the hydrophilic block [17]. Amphiphilic compounds containing highly hydrophobic cholesteryl groups can associate in water through hydrophobic interactions [18]. Polymers containing cholesteryl groups form hierarchically stable nanostructures by self-assembly upon heating [19,20,21,22]. Amphipathic block copolymers containing cholesteryl moieties adopt a liquid crystalline phase from disk-shaped micelles and a stacked-disk morphology in water [23,24].

Thermo-responsive poly(*N*-isopropyl acrylamide) (PNIPAM) exhibits a lower critical solution temperature (LCST) in water [25,26,27,28]. PNIPAM can dissolve in water below the LCST because the pendant amide groups are hydrated through hydrogen bonding interactions with water molecules. Above the LCST, the pendant amide groups are dehydrated because of the increased molecular motion to destroy the hydrogen bonding interactions with water molecules, and PNIPAM cannot dissolve in water. The thermo-response behavior of PNIPAM has been applied in the fields of medicine, pharmacy, and engineering [29]. Boissé et al. [30]. reported the association behavior of amphipathic diblock copolymers composed of the hydrophobic cholesteryl groups-containing block exhibiting liquid crystallinity and the thermo-responsive poly(*N*,*N*-diethylacrylamide) (PDEA) block. The amphipathic diblock copolymers form fibers with a smectic ordered structure because of the mesogenic cholesteryl groups in water. Above the LCST, PDEA on the fiber surface became hydrophobic and aggregated to form a precipitate.

**Figure 1:**
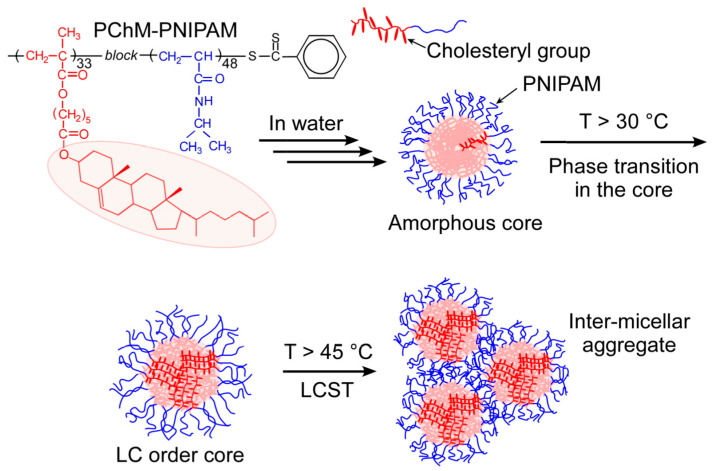
Chemical structure of PChM-PNIPAM and conceptual illustration of aggregation and thermo-responsive behaviors of PChM-PNIPAM in water.

In this study, a diblock copolymer (PChM-PNIPAM) composed of the hydrophobic liquid crystalline poly(cholesteryl 6-methacryloyloxy hexanoate) (PChM) block and the thermo-responsive PNIPAM block was prepared via reversible addition–fragmentation chain transfer (RAFT) radical polymerization (Figure 1). In water, PChM-PNIPAM formed polymer micelles with a hydrophobic PChM block core and hydrophilic PNIPMAM shells below the LCST. Increasing the temperature of the PChM-PNIPAM aqueous solution induced a liquid crystalline phase transition of the pendant cholesteryl groups in the core. Further increases in temperature above the LCST resulted in dehydration of the PNIPAM shells and agglomeration to form large inter-micellar aggregates. We have studied the self-association and the thermo-responsive behaviors of PChM-PNIPAM in water.

## 2. Experimental

### 2.1. Materials

Cholesterol (90%), methacrylic acid (>99.0%), hydrochloric acid (35–37%), sodium sulfate (99.0%), tetrabutylammonium bromide (>98.0%), 6-bromohexanoyl chloride (97.0%), potassium hydroxide (85.0%), ethanol (99.5%), tetrahydrofuran (THF, 99.9%), and 2,2′-azobis(4-methoxy-2,4-dimethylvaleronitrile) (V-70, 95.0%) from Fujifilm Wako Pure Chemical (Osaka, Japan) were used without purification. 2,6-Di-*t*-butylcresol (98.0%) was obtained from Kanto Chemical (Tokyo, Japan). Diethyl ether (99%) was acquired from Hayashi Pure Chemical (Osaka, Japan). Methanol (99.9%) was supplied by KT Chemicals (Osaka, Japan) and used as received. 2,2′-Azobis(isobutyronitrile) (AIBN, 98%) from Fujifilm Wako Pure Chemical (Osaka, Japan) was recrystallized from methanol. Potassium methacrylate was prepared from a reaction between methacrylic acid (77.5 g, 0.900 mol) and potassium hydroxide (50.5 g, 0.900 mol) in methanol. The reaction mixture was poured into diethyl ether to precipitate. The precipitate was dried under reduced pressure to obtain potassium methacrylate (89.6 g, 80.2%). Pyridine (99.5%) from Fujifilm Wako Pure Chemical (Osaka, Japan) was dried in molecular sieves 4 Å for one day before use. Chloroform (≥98.0%) from Nacalai Tesque (Kyoto, Japan) was treated with activated alumina for one day before being used to remove ethanol. 4-Cyanopentanoate dithiobenzoate (CPD) was prepared using the methodology reported elsewhere [31]. *N*-Isopropylacrylamide (NIPAM) gifted from KJ Chemicals (Tokyo, Japan) was recrystallized from *n*-hexane. Pyrene (97.0%) from Fujifilm Wako Pure Chemical (Osaka, Japan) was recrystallized from methanol. α-Methyl trithiocarbonate-*S*-phenylacetic acid (MTPA) was prepared using the methodology reported elsewhere [32]. Water was purified using an ion-exchange column system. Dextran and Texas Red–labeled dextran (MW 3000, TD) were purchased from Molecular Probes (Eugene, OR, USA) and used as received. PNIPAM was prepared via RAFT polymerization using MTPA as a chain transfer agent (CTA). The number-average molecular weight (*M*_n_), degree of polymerization (DP), and molecular weight distribution (*M*_w_/*M*_n_) values of PNIPAM were 6.00 × 10^3^ g/mol, 53, and 1.15, respectively, as measured by gel-permeation chromatography (GPC) [33].

### 2.2. Preparation of Cholesteryl 6-(Methacryloyloxy)Hexanoate (ChM)

Cholesteryl 6-(methacryloyloxy)hexanoate (ChM) was prepared using a two-step reaction (Scheme S1) [34]. First, cholesteryl 6-bromohexanoate was synthesized. A chloroform (50.0 mL) solution of 6-bromohexanoyl chloride (23.7 g, 0.111 mol) was poured into a chloroform (200 mL) solution of cholesterol (34.6 g, 0.0896 mol) and pyridine (8.50 g, 0.107 mol) at 0 °C over a 30 min period. After stirring for 2 h at 0 °C and then 16 h at room temperature, the mixture was washed twice with 1.0 N hydrochloric acid (200 mL) and once with pure water. After drying the organic layer over sodium sulfate, the solvent was evaporated. The solid was recrystallized twice using a mixture of diethyl ether and ethanol. After purification, cholesteryl 6-bromohexanoate was dried under reduced pressure (22.5 g, 44.6%); mp 120–121 °C; ^1^H nuclear magnetic resonance (NMR) (CDCl_3_, δ): 5.38 (m, 1H), 4.60 (m, 1H), 3.39 (t, 2H), 2.30 (m, 4H), 2.10–0.65 (m, 45H). Cholesteryl 6-bromohexanoate (20.0 g, 35.5 mmol) and 2,6-di-*t*-butyl cresol (0.527 g, 2.39 mmol) were dissolved in chloroform (97.0 mL). Potassium methacrylate (13.3 g, 107 mmol) and tetrabutylammonium bromide (2.25 g, 6.97 mmol) were dissolved separately in water (48.0 mL), and the two solutions were mixed. The mixture was heated under reflux at 110 °C for 40 h. After the reaction, the mixture was diluted with diethyl ether and chloroform (500 mL, 4/1; *v*/*v*). The solution was washed twice with pure water and once with brine and then dried over sodium sulfate. After evaporating the solvent, the product was recrystallized using methanol/ether. The resulting crystals (ChM) were dried under vacuum (13.7 g, 68.3%); mp 56.0–58.1 °C; ^1^H NMR (CDCl_3_, δ): 6.08 (bs, 1H), 5.54 (bs, 1H), 5.38 (m, 1H), 4.60 (m, 1H), 4.13 (t, 2H), 2.30 (m, 4H), 2.1–0.65 (m, 50H).

### 2.3. Preparation of PChM and PChM-PNIPAM

PChM was synthesized via RAFT polymerization (Scheme S2). ChM (1.66 g, 2.95 mmol), CPD (20.9 mg, 74.9 μmol), and V-70 (9.91 mg, 32.1 μmol) were dissolved in THF (3.0 mL). The feed molar ratio of [ChM]/[CPD]/[V-70] was 39/1/0.4. The solution was heated to 40 °C for 24 h under Ar atmosphere. The conversion of ChM was determined to be 90.1% by ^1^H NMR spectroscopy. The polymerization mixture was dialyzed against THF for one day and precipitated using methanol. The precipitate (PChM) was dried under vacuum overnight at 40 °C (843 mg, 50.8%). The DP was 33, as estimated from the ^1^H NMR spectrum. The *M*_n_ and *M*_w_/*M*_n_ values obtained from the GPC measurements were 2.34 × 10^4^ g/mol and 1.13, respectively.

PChM-PNIPAM was prepared via RAFT polymerization using PChM macro-CTA. NIPAM (120 mg, 1.06 mmol), PChM (198 mg, 10.5 µmol), and V-70 (1.60 mg, 5.19 μmol) were dissolved in THF (1.38 mL). The molar feed ratio of [ChM]/[CPD]/[V-70] was 101/1/0.5. The solution was heated to 40 °C for 18 h under Ar atmosphere. The conversion of NIPAM was determined to be 49.5% according to ^1^H NMR spectroscopy. The polymerization solution was poured into methanol to precipitate PChM-PNIPAM. PChM-PNIPAM was collected and dried under vacuum overnight at 40 °C (102 mg, 32.0%). The DP of the PNIPAM block was 48, as estimated from the ^1^H NMR. The *M*_n_ and *M*_w_/*M*_n_ values obtained from GPC were 2.42 × 10^4^ g/mol and 1.12, respectively.

### 2.4. Preparation of PChM-PNIPAM Aqueous Solution

PChM-PNIPAM (5.60 mg, 0.230 µmol) was dissolved in THF (5.65 mL) with a polymer concentration (*C*_p_) of 0.991 g/L. The THF solution was dialyzed against pure water for two days to prepare the aqueous polymer solution. The aqueous polymer solution was diluted with water to *C*_p_ = 0.04 g/L.

### 2.5. Measurements

^1^H NMR spectroscopy (DRX-500, Bruker, Billerica, MA, USA) was performed at 25 °C. Fourier-transform infrared (FTIR FT/IR-4200) spectroscopy was performed using the potassium bromide (KBr) pellet method. The GPC measurements were performed at 40 °C using a Shodex (Tokyo, Japan) DS-4 pump, a GF-7M column, and a RI-101 refractive index detector. THF was used as the developing solvent at a flow rate of 1.0 mL/min. *M*_n_ and *M*_w_/*M*_n_ were determined in terms of standard polystyrene. The melting point and liquid crystal phase transition temperature were measured using a Yanaco (Kyoto, Japan) MP-J3 melting point apparatus. Differential scanning calorimetry (DSC, DSC7000X, Hitachi, Tokyo, Japan) was performed in the range of 20–230 °C at a heating rate of 1.0 °C/min. The phase transition temperatures were determined using a Hitachi (Tokyo, Japan) easy and multipurpose analysis software version 1.0.0. Dynamic light scattering (DLS, Zetasizer Nano ZS, Malvern, UK) was measured at a scattering angle of 173°. The hydrodynamic radius (*R*_h_) and light scattering intensity (LSI) of the aqueous polymer solutions were monitored in the range of 10–70 °C. Static light scattering (SLS, DLS-7000, Otsuka Electronics, Osaka, Japan) was performed at 25 °C using a He–Ne laser (632.8 nm, 10.0 mW) as the light source. The weight-average molecular weight (*M*_w_) was calculated from the Zimm plot. The refractive index as a function of the polymer concentration (d*n*/d*C*_p_) at 633 nm was determined using an Otsuka Electronics (Osaka, Japan) DRM-3000 differential refractometer at 25 °C. The fluorescence measurements were performed using a Hitachi (Tokyo, Japan) F-2500 fluorescence spectrophotometer. The polymer was dissolved in a saturated aqueous pyrene solution (6.0 × 10^−7^ M). The solution was excited at 334 nm, and the excitation and emission slit widths were measured at 20 and 2.5 nm, respectively. Scanning electron microscopy (SEM) was performed using a Keyence (Osaka, Japan) VE-9800 microscope. The sample was prepared by dropping aqueous sample solutions on Al stages at 25 and 70 °C on a hot plate. The sample was dried in air at 25 and 70 °C and sputtered with platinum. Transmission electron microscopy (TEM, JEM-2100, JEOL, Tokyo, Japan) was performed at an acceleration voltage of 160 kV. The samples were prepared by placing a drop of aqueous sample solution on a carbon-coated copper grid at 25 and 70 °C on a hot plate. The samples were then stained with an aqueous sodium phosphotungstate solution and dried for one day under reduced pressure. The percentage transmittance (%*T*) at 700 nm of the aqueous polymer solutions was monitored in the range of 10–80 °C with 1.0 °C/min using a Jasco (Tokyo, Japan) V-630BIO UV-vis spectrophotometer. Small-angle X-ray scattering (SAXS) measurements were taken at the SPring-8 BL40B2 beamline with an X-ray wavelength of 0.1 nm, a camera length of 1 m, a capillary cell with a thickness of 2 mm, and a PILATUS 2M detector. The irradiation time was 3 min. The measurements were taken after waiting for 1 min after the solution temperature reached each temperature.

## 3. Results and Discussion

### 3.1. Polymer Characterization

**Figure 2:**
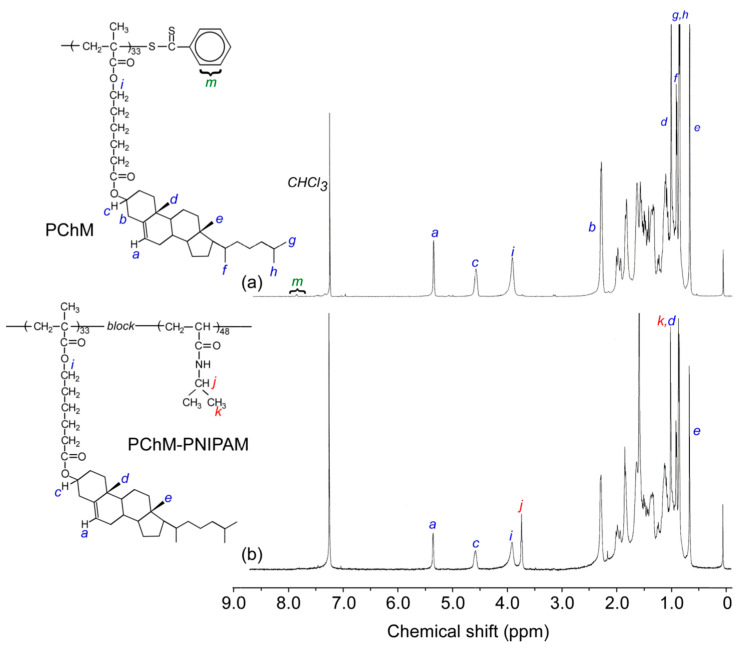
^1^H NMR spectra of (**a**) PChM and (**b**) PChM-PNIPAM in CDCl_3_.

The chemical structures of cholesterol, cholesteryl 6-bromohexanoate, and ChM were confirmed by ^1^H NMR (Figure S1). PChM-PNIPAM was prepared to polymerize NIPAM using PChM macro-CTA via RAFT. The ^1^H NMR spectra of PChM and PChM-PNIPAM in CDCl_3_ were then measured (Figure 2). The calculated DP(NMR) of PChM was 33 from the integrated intensity ratio of the double bond proton signal of the pendant cholesteryl groups in the PChM block at 5.4 ppm (*a*) and the phenyl proton signal of the dithiobenzoate group at the polymer chain end around 7.7 ppm (*m*). The *M*_n_(NMR) of PChM estimated from the NMR spectrum was 1.89 × 10^4^ g/mol. The DP(NMR) of the PNIPAM block in PChM-PNIPAM was calculated to be 48 from the integrated intensity ratio of the pendant cholesteryl groups in the PChM block at 5.4 ppm (*a*) and the methine proton signal of the pendant isopropyl groups in the PNIPAM block at 3.7 ppm (*j*). The integral intensity ratio of peak *a* and *j* was 33/48 for PChM-PNIPAM. The *M*_n_(NMR) of PChM-PNIPAM obtained by NMR spectroscopy was 2.43 × 10^4^ g/mol.

The theoretical DP (DP(theo)) and theoretical molecular weight (*M*_n_(theo)) were calculated using the following equations:

(1) $$\mathrm{DP}\left(\mathrm{theo}\right)=\frac{{ [M]}_{0}}{{ [\mathrm{CTA}]}_{0}}\times \frac{x}{100}$$

(2) $$M_{n}\left(\mathrm{theo}\right)=\mathrm{DP}\left(\mathrm{theo}\right)\times M_{m}+M_{\mathrm{CTA}}$$

where [M]_0_ and [CTA]_0_ are the initial concentrations of the monomer and CTA, respectively; *x* is the percentage conversion obtained from the ^1^H NMR spectra after polymerization; and *M*_m_ and *M*_CTA_ are the molecular weights of the monomer and CTA, respectively. The *M*_n_(theo) values of PChM and PChM-PNIPAM were 2.05 × 10^4^ g/mol and 2.45 × 10^4^ g/mol, respectively (Table 1). GPC was measured for PChM and PChM-PNIPAM using THF as the eluent (Figure S2). Both GPC elution curves were similar, while the peak for PChM-PNIPAM was shifted to a higher molecular weight region compared with that for PChM. Conversely, the *M*_n_(GPC) values were similar for each polymer, presumably because there were unexpected interactions between the polymer and the GPC column [35]. The *M*_n_(GPC) values for PChM and PChM-PNIPAM were 2.34 × 10^4^ g/mol and 2.42 × 10^4^ g/mol, respectively. The *M*_n_(NMR) values were close to the *M*_n_(theo) values. The *M*_w_/*M*_n_ values for PChM and PChM-PNIPAM were narrow at 1.13 and 1.12, respectively. These observations suggested that the polymer structures were well controlled.

For comparison, PNIPAM with DP = 53 was prepared via RAFT. The FTIR spectra of PNIPAM with DP = 53, PChM, and PChM-PNIPAM were measured (Figure S3). The amide-stretching band at 1650 cm^−1^ was observed for PNIPAM. PChM showed a stretching band for an ester at 1720 cm^−1^. The FTIR spectrum of PChM-PNIPAM showed both peaks at 1650 and 1720 cm^−1^. The glass transition temperature (*T*_g_) and melting point (*T*_m_) were examined by second heating DSC cycles for PChM and PChM-PNIPAM (Table S1 and Figure S4). For PChM, the *T*_g_ was observed at 51 °C. An endothermic peak associated with *T*_m_ from the liquid crystalline to anisotropic phases was observed at 169 °C [36]. When PChM was observed using crossed Nicols of a polarizing optical microscope with heating, a bright field was noted above 51 °C, and a liquid crystalline texture was confirmed. The observation field became dark with further increases in temperature above 169 °C, and a transition from a liquid crystalline phase to an isotropic phase was observed. From PChM-PNIPAM, the *T*_g_ of the PChM and PNIPAM blocks were 50 °C and 140 °C, respectively [37]. Furthermore, the *T*_m_ associated with the transition from liquid crystal to isotropic phases was observed as an endothermic peak. In the crossed Nicols observation for PChM-PNIPAM, the bright field with a liquid crystalline texture was observed above 50 °C, and the observation field was dark above 171 °C because of a transition from liquid crystalline to isotropic phases. DSC of PChM and PChM-PNIPAM revealed similar *T*_g_ and *T*_m_ values.

### 3.2. Self-Association Behavior of PChM-PNIPAM in Water

**Figure 3:**
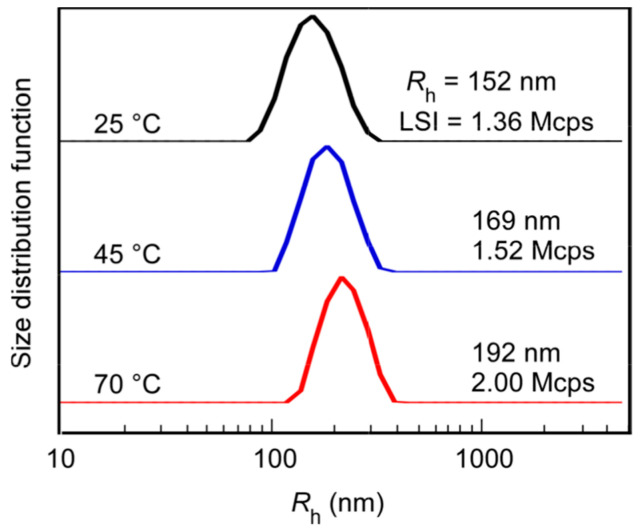
Hydrodynamic radius (*R*_h_) distributions for PChM-PNIPAM in water with polymer concentration (*C*_p_) = 0.04 g/L at 25 °C, 45 °C, and 70 °C.

The association behavior of PChM-PNIPAM in water at 25 °C was examined by DLS (Figure 3). A unimodal *R*_h_ distribution was observed with *R*_h_ = 152 nm. The pendant amide groups in the PNIPAM block were hydrated at 25 °C which was lower than the LCST. The PChM block was associated to form the core surrounded by hydrophilic PNIPAM shells to form polymer micelles. At 45 °C and 70 °C, the *R*_h_ values increased to 169 nm and 192 nm, respectively. This result suggests that a two-stage change occurs with increasing temperature. Further details are discussed in the next section.

SLS was measured to examine the association behavior of PChM-PNIPAM in water at 25 °C in more detail (Figure S5). The d*n*/d*C*_p_ was 0.101 mL/g, as determined using a differential refractometer, which is needed to determine the apparent weight-average molecular weight (*M*_w_(SLS)). The apparent *M*_w_(SLS) of the polymer micelle obtained from SLS was 7.73 × 10^9^ g/mol (Table 2). The aggregate number (*N*_agg_), which is the number of polymer chains forming a single micelle, was calculated from the following equation: *N*_agg_ = *M*_w_(SLS)/(*M*_n_(NMR) × *M*_w_/*M*_n_) [38]. The *N*_agg_ of PChM-PNIPAM in water was 2.84 × 10^5^. The radius of gyration (*R*_g_) obtained from SLS was 114 nm. The *R*_g_/*R*_h_ of the aggregate was 0.75, which is close to that of a rigid sphere [39]. The density (Φ_H_) of the micelle was calculated using the following equation:

(3) $$\Phi_{H}=\frac{M_{w}\left(\mathrm{SLS}\right)}{N_{A}}{\left(\frac{4}{3}\pi R_{h}{}^{3}\right)}^{-1}$$

where *N*_A_ is Avogadro’s number. The Φ_H_ of the polymer micelle formed from PChM-PNIPAM in water was calculated to be 0.873 g/cm^3^. The Φ_H_ of the aggregate formed from dextran with randomly bearing hydrophobic cholesteryl groups in water was 0.017–0.109 g/cm^3^ [40]. The Φ_H_ of the PChM-PNIPAM micelle was larger than those of cholesterol-bearing dextran because the block copolymer structure formed the highly dehydrated core with the PChM blocks in water. The *R*_g_/*R*_h_ = 0.75, which is close to a rigid sphere, supports the high Φ_H_ of the PChM-PNIPAM micelles.

**Figure 4:**
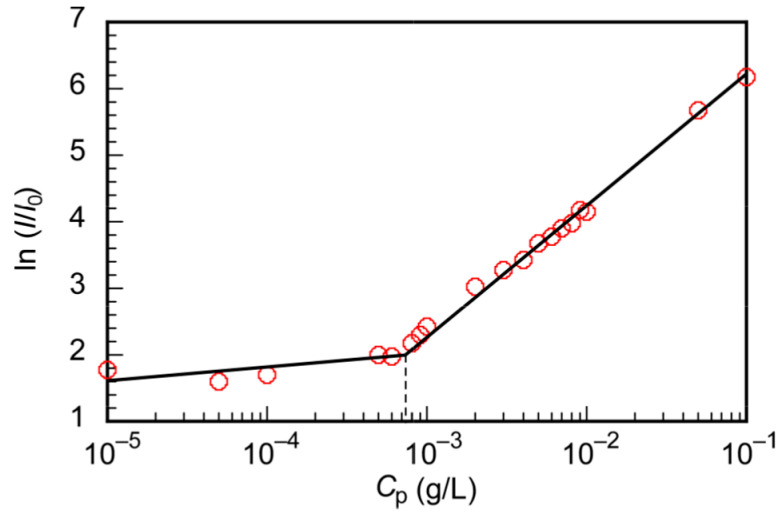
Light scattering intensity ratio (*I*/*I*_0_) of an aqueous PChM-PNIPAM solution as a function of the polymer concentration (*C*_p_). *I* and *I*_0_ are scattering intensities of the polymer solution and water at 25 °C, respectively.

The light scattering intensity (LSI) depends on the size of the aggregates. The LSI decreases when the aggregates dissociate. The critical micelle concentration (CMC) was determined from a plot of the LSI ratio (*I*/*I*_0_) of the solution (*I*) to the solvent (*I*_0_) as a function of the polymer concentration (*C*_p_) of PChM-PNIPAM (Figure 4). The CMC(LSI) was estimated to be 7.2 × 10^−4^ g/L from the inflection point of the plot. The CMC of the amphipathic diblock copolymer (PS-PEO) with polystyrene and polyethylene glycol was reported to be 1.6 × 10^−3^ g/L [41]. The CMC of PChM-PNIPAM was smaller than that of PS-PEO because the PChM block was more hydrophobic than polystyrene.

**Figure 5:**
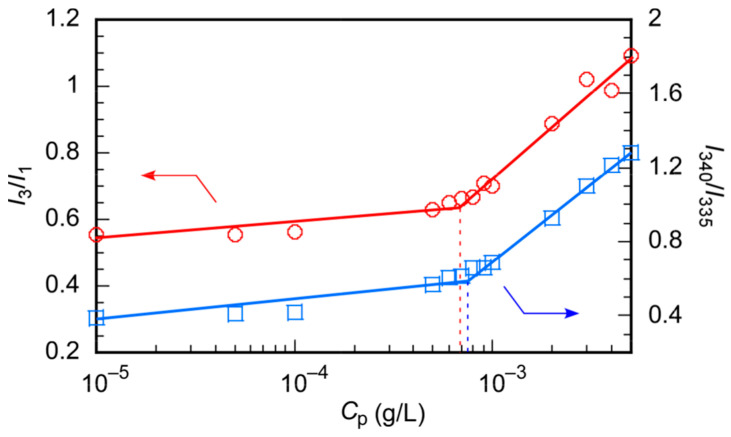
*I*_3_/*I*_1_ (red circle) of pyrene fluorescence and *I*_340_/*I*_335_ (blue square) of pyrene excitation spectra in the presence of PChM-PNIPAM as a function of the polymer concentration (*C*_p_) at 25 °C; *I*_3_ and *I*_1_ are the fluorescence vibronic peak intensities at 1 and 3, and *I*_340_ and *I*_335_ are the emission intensities at 340 and 335 nm of the pyrene excitation spectra, respectively.

The CMC of PChM-PNIPAM was determined using pyrene as a fluorescence probe in water (Figure 5). The emission intensity ratio (*I*_3_/*I*_1_) of the first vibration peak at 373 nm (*I*_1_) and the third vibration peak at 385 nm (*I*_3_) in the pyrene fluorescence spectra depends on the microenvironmental polarity around the pyrene molecule [42]. *I*_3_/*I*_1_ increases because pyrene exists in a hydrophobic environment. *I*_3_/*I*_1_ vs. *C*_p_ of PChM-PNIPAM was plotted, and CMC(Em) was estimated from the inflection point. As the microenvironmental polarity around pyrene becomes hydrophobic, the 0-0 band maximum wavelength in the pyrene excitation spectra shifts to a longer wavelength [43]. The maximum wavelength of the 0-0 band in water was 335 nm, but it shifted to a longer wavelength at 338 nm in the presence of 0.1 g/L PChM-PNIPAM. This suggests that pyrene was incorporated into the hydrophobic domain formed from PChM-PNIPAM. The ratio of the fluorescence intensities at wavelengths of 338 and 335 nm (*I*_338_/*I*_335_) was plotted as a function of *C*_p_ to estimate the CMC(Ex) from the inflection point. The CMC(Em) and CMC(Ex) obtained from the pyrene fluorescence and excitation spectra were 7.0 × 10^−4^ g/L and 7.2 × 10^−4^ g/L, respectively. These values were similar to 7.2 × 10^−4^ g/L of CMC(LSI) obtained from the LSI experiment.

**Figure 6:**
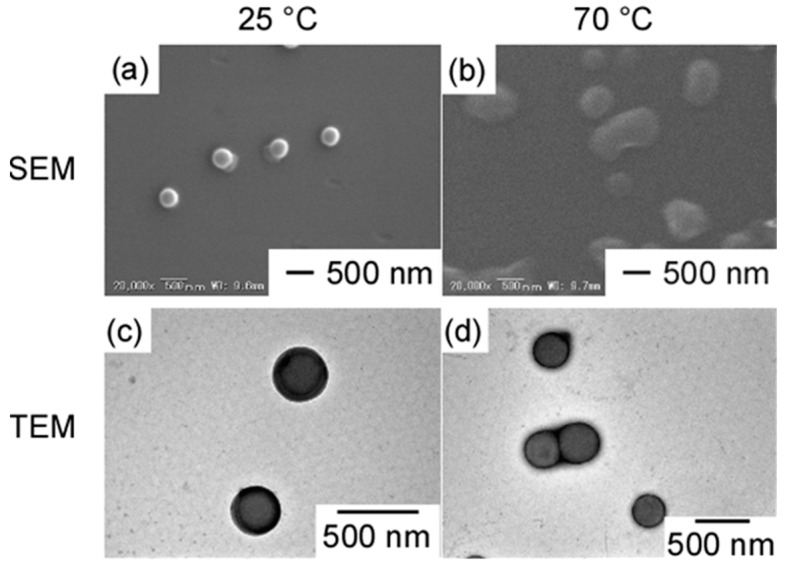
SEM images of PChM-PNIPAM at (**a**) 25 °C and (**b**) 70 °C, and TEM images of PChM-PNIPAM at (**c**) 25 °C and (**d**) 70 °C.

SEM and TEM samples were prepared using an aqueous solution of PChM-PNIPAM at 25 °C. For the SEM sample, a drop of the solution was placed on a temperature-controlled aluminum stage, and excess water was absorbed with filter paper. The sample was then sputtered with platinum. For the TEM sample, a drop of the solution was placed on a temperature-controlled TEM grid at 25 °C, with the excess water absorbed using filter paper, followed by staining with sodium phosphotungstate. All processes were conducted at 25 °C. SEM and TEM revealed spherical aggregates with a uniform size (Figure 6a,c). The average radii obtained from SEM (*R*_SEM_) and TEM (*R*_TEM_) were 159 and 163 nm, respectively. These values were similar to *R*_h_ (=152 nm) of the aggregate in water at 25 °C. The contrast of the spherical aggregate inside was lower than that of outside, according to TEM. A strongly stained outside or formed vesicle structure may be responsible. If the aggregate was a vesicle, hydrophilic guest molecules could be incorporated into the hollow core. The fluorescence-labeled hydrophilic guest molecule, Texas Red Dextran (TD), was used to incorporate the PChM-PNIPAM aggregates. PChM-PNIPAM and TD were dissolved in THF, which was dialyzed against water. The TD THF solution without PChM-PNIPAM was dialyzed against water as a control. No fluorescence from TD was observed from the aqueous solution in the dialysis membrane after dialysis for 13 days. All the TD permeated through the dialysis membrane regardless of the presence of PChM-PNIPAM. PChM-PNIPAM aggregates cannot incorporate hydrophilic TD molecules. Therefore, the PChM-PNIPAM aggregates in water existed as a solid particle like a core–shell spherical polymer micelle.

### 3.3. Thermo-Responsive Behavior of PChM-PNIPAM

The percentage transmittance (%*T*) at 700 nm of the PChM-PNIPAM aqueous solution was plotted as a function of temperature (Figure S6). The %*T* was kept constant at approximately 89% during the heating and cooling processes, and no clear transition temperature was observed. PChM-PNIPAM formed interpolymer aggregates with a size of <700 nm, which did not affect %*T* at 700 nm.

**Figure 7:**
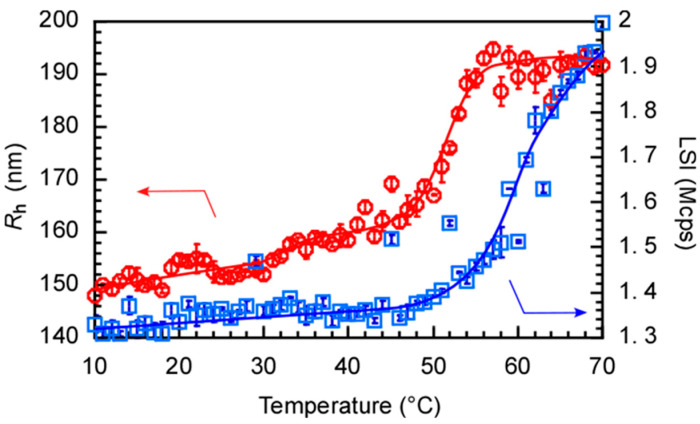
Hydrodynamic radius (*R*_h_, red circle) and light scattering intensity (LSI, blue square) of PChM-PNIPAM in water with *C*_p_ = 0.04 g/L as a function of temperature.

The *R*_h_ and LSI values for the PChM-PNIPAM aqueous solution were measured at varying temperatures (Figure 7). PChM-PNIPAM formed polymer micelles with *R*_h_ = 152 nm at 25 °C. *R*_h_ increased in two steps with increasing temperature. *R*_h_ increased to 159 nm above 34 °C and increased further above 45 °C, reaching 192 nm at 70 °C. The *R*_h_ distributions at 45 and 70 °C were unimodal (Figure 3). In the first *R*_h_ increasing step at 34 °C, the *N*_agg_ of the aggregates was constant because of the constant LSI around 34 °C. Therefore, the pendant cholesteryl groups in the PChM block were transferred from the amorphous-to-liquid crystal phases in the core. A slight increase in *R*_h_ was observed because the core was expanded during the amorphous-to-liquid crystalline phase transition. The LSI is strongly affected by particle size, molecular weight, and density. The LSI began to increase above 45 °C. The increase in *R*_h_ in the second step above 45 °C was attributed to inter-micellar aggregation caused by the hydrophobic PNIPAM shells above the LCST. A PNIPAM homopolymer with DP = 53 was prepared. The DP was close to 48 in the NIPAM block of PChM-PNIPAM. The LCST for PNIPAM with DP = 53 was 45 °C with the heating process (Figure S7). Therefore, the increase in *R*_h_ at 45 °C for the aqueous PChM-PNIPAM solution was attributed to the LCST of the PNIPAM block.

The SEM and TEM samples were prepared at 70 °C (Figure 6b,d), which revealed interparticle aggregates. *R*_SEM_ and *R*_TEM_ were 339 and 171 nm, respectively. Interparticle aggregates were formed because the temperature at 70 °C was higher than the LCST of the PNIPAM shells.

**Figure 8:**
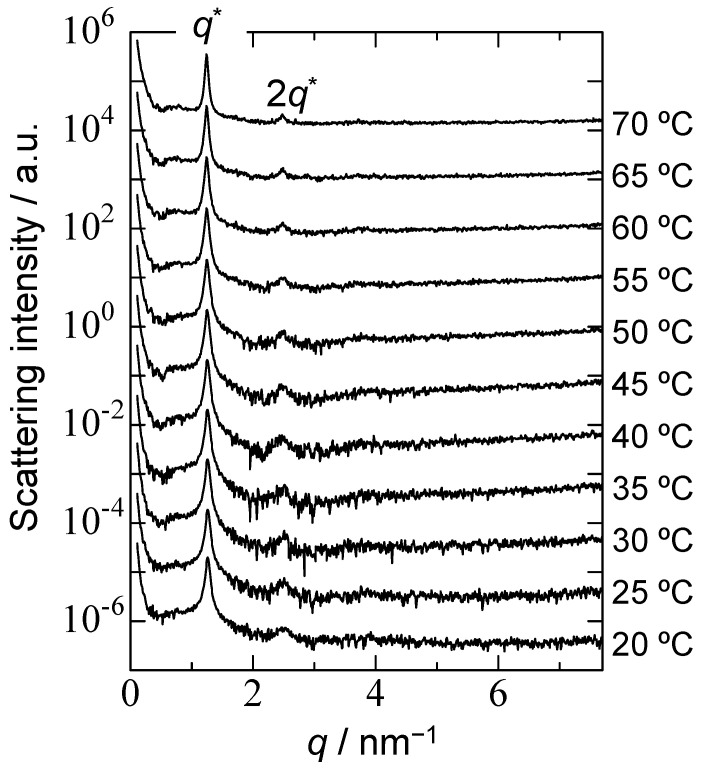
Small angle X-ray scattering (SAXS) patterns of PChM-PNIPAM in water with *C*_p_ = 1.0 g/L at varying temperatures. *q* is the magnitude of the scattering vector and q* is the primary-order peak.

Light scattering and %*T* were measured at 0.04 g/L because the solution became turbid at high concentrations. Conversely, small angle X-ray scattering (SAXS) of the diluted sample with *C*_p_ = 0.04 g/L could not produce good data because of a poor S/N ratio. Therefore, the PChM-PNIPAM aqueous solution with *C*_p_ = 0.04 g/L was concentrated to 1.0 g/L and then measured by SAXS (Figure 8). At 20 °C, peaks attributed to the orientation of the pendant cholesteryl groups in the core were observed. The pendant cholesteryl groups in the core were ordered presumably because of heating to increase the *C*_p_ by evaporation. The ordered structure as a liquid crystal phase in the core was maintained as a crystalline phase, even when cooled to 20 °C. The ordered liquid crystalline structure was frozen as a crystalline ordered structure. The PChM-PNIPAM aqueous solution was prepared using a dialysis method from the THF solution against pure water to form aggregates caused by hydrophobic interactions. The pendant cholesteryl groups aggregated in an amorphous state when the hydrophobic PChM blocks formed the core. The core became a liquid crystalline state upon heating, and the order of the cholesteryl groups remained, even when the temperature was decreased to 20 °C. Two XRD peaks were observed from the SAXS data at all temperatures. The magnitude of the scattering vectors (*q*) of the primary (q*) and secondary order (2q*) peaks were 1.25 and 2.50 nm^−1^, respectively. This suggests that the cholesteryl group forms a lamellar structure in the core [44]. The lattice spacing (*d*) was calculated from the following equation: *d* = 2*π*/*q*. The *d* obtained from the primary-order peak was 5.03 nm. This *d* value corresponded to the thickness of the lamellar structure when the cholesteryl groups in the PChM were oriented as a two-layer packing structure (Figure S8) [45]. At 70 °C, the primary and secondary peaks assigned to the ordered structure of the PChM-PNIPAM aqueous solutions remained. The *T*_m_ of bulk PChM- PNIPAM was 171 °C (Figure S4). The orientation of the cholesterol groups in the core in water was maintained at 70 °C. The cholesteryl groups in the core of the polymer micelles formed from PChM-PNIPAM in water were tightly packed because of strong hydrophobic interactions of the PChM blocks. Therefore, the Φ_H_ was as large as 0.873 g/cm^3^, and *R*_g_/*R*_h_ suggested a rigid spherical structure. The core formed an amorphous state when the PChM-PNIPAM aqueous solution was prepared using a dialysis method. Above 34 °C, the amorphous core changed to a liquid crystalline phase because of the increased motion of molecules in the core upon heating. The orientation of cholesteryl groups in the core was frozen as a glass state when the temperature was below *T*_g_ in water.

## 4. Conclusions

PChM-PNIPAM, composed of PChM exhibiting hydrophobic liquid crystalline behavior and PNIPAM with an LCST in water, was prepared via RAFT polymerization. In water at 25 °C, PChM-PNIPAM formed spherical polymer micelles with *R*_h_ = 152 nm because of hydrophobic interactions. The *N*_agg_ and Φ_H_ were 2.84 × 10^5^ and 0.873 g/cm^3^, respectively. These high values suggested that PChM-PNIPAM formed dense-packed aggregates. Furthermore, the *R*_g_/*R*_h_ indicated a rigid sphere. SEM and TEM revealed spherical objects. The PChM-PNIPAM aqueous solution increased *R*_h_ in two steps by heating. *R*_h_ increased from 152 to 169 nm at temperatures above 34 °C, with further increases above 45 °C, reaching 192 nm at 70 °C. The first increase in *R*_h_ suggests an increase in the core size due to the phase transition of the cholesteryl groups in the core from the amorphous to the liquid crystalline phase. In the second stage, the increase in *R*_h_ indicated interparticle aggregation caused by dehydration of the PNIPAM shells. The SAXS data suggested that the pendant cholesteryl groups in the core underwent self-assembly to form lamellar structures. When PChM-PNIPAM was heated in water, the core changed from an amorphous to a liquid crystalline state. Conversely, after cooling from the liquid crystalline state, the order of the liquid crystalline state in the core was kept as a frozen aggregate in the crystal state at room temperature. The thermo-responsive polymer micelles have potential applications as carriers that regulate guest molecule release in accordance with phase transition from the liquid crystalline to the amorphous phase.

## Figures and Tables

**Table 1:** Characteristics of PChM and PChM-PNIPAM.

Sample	DP(theo) ^a^	*M*_n_(theo) ^b^ × 10^−4^ (g/mol)	DP(NMR) ^c^	*M*_n_(NMR) ^c^ × 10^−4^ (g/mol)	DP(GPC) ^d^	*M*_n_(GPC) ^d^ × 10^−4^ (g/mol)	*M*_w_/*M*_n_^d^
PChM	36	2.05	33	1.89	41	2.34	1.13
PChM-PNIPAM	50 ^a^	2.45	48 ^a^	2.43	47 ^a^	2.42	1.12

^a^ Calculated from Equation (1). ^b^ Calculated from Equation (2). ^c^ Estimated from the ^1^H NMR spectra. ^d^ Estimated from GPC. Abbreviations: PChM, poly(cholesteryl 6-methacryloyloxy hexanoate); PChM-PNIPAM, poly(cholesteryl 6-methacryloyloxy hexanoate)-*block*-poly(*N*-isopropyl acrylamide); DP, degree of polymerization; *M*_n_, number-average molecular weight.

**Table 2:** Characteristics of PChM-PNIPAM micelles in water at 25 °C.

*M*_w_(SLS) ^a^ (g/mol)	*N*_agg_^b^	*R*_h_^c^ (nm)	*R*_g_^d^ (nm)	*R*_g_/*R*_h_	Φ_H_^e^ (g/cm^3^)
7.73 × 10^9^	2.84 × 10^5^	152	114	0.75	0.873

^a^ Apparent weight average molecular weight (*M*_w_(SLS)) estimated from static light scattering (SLS). ^b^ Aggregation number (*N*_agg_). ^c^ Hydrodynamic radius (*R*_h_) estimated by DLS. ^d^ Radius of gyration (*R*_g_) estimated from SLS. ^e^ Density of the polymer aggregate estimated from Equation (3).

## Data Availability

Data are contained within the article or Supplementary Material.

## Supplementary Materials

The following supporting information can be downloaded at: https://www.mdpi.com/article/10.3390/polym15030770/s1, Table S1: Glass transition (*T*_g_) and melting temperatures (*T*_m_) of PChM and PChM-PNIPAM; Scheme S1: Synthesis of ChM; Scheme S2. Synthesis of (a) PChM and (b) PChM-PNIPAM; Figure S1: ^1^H NMR spectra of (a) cholesterol, (b) cholesteryl 6-bromohexanoate, and (c) ChM in CDCl_3_; Figure S2: Gel-permeation chromatography (GPC) elution curve for PChM (---) and PChM-PNIPAM (**—**) using THF as the eluent with a flow rate of 1.0 mL/min at 40 °C; Figure S3: Fourier-transform infrared (FTIR) spectra using KBr pellets for (a) PNIPAM, (b) PChM, and (c) PChM-PNIPAM; Figure S4: Differential scanning calorimetry (DSC) curves of (a) PChM and (b) PChM-PNIPAM with the second heating processes. The inserts are typical polarizing optical micrographs for (a) PChM at 140 °C and (b) PChM-PNIPAM at 110 °C; Figure S5: Zimm plot for PChM-PNIPAM in water at 25 °C; Figure S6: Percentage transmittance (%*T*) for the PChM-PNIPAM aqueous solution with a polymer concentration (*C*_p_) = 0.04 g/L as a function of temperature: heating (**―**) and cooling processes (---); Figure S7: Percentage transmittance (%*T*) of the PNIPAM_53_ with degree of polymerization (DP) = 53 and aqueous solution with polymer concentration (*C*_p_) = 0.05 g/L as a function of temperature: heating (**―**) and cooling processes (---); Figure S8: Conceptual illustration of the packing structures of the pendant cholesteryl groups of PChM-PNIPAM in an aqueous solution.
