# Supplementary material for: A Thermo-Responsive Polymer Micelle with a Liquid Crystalline Core
Source: Polymers (Basel). 2023 Feb 2;15(3):770. doi: 10.3390/polym15030770 (PMC9920352; doi:10.3390/polym15030770)
Supplement: Supplementary file 1 [file polymers-15-00770-s001.zip › polymers-2194998-supplementary.pdf]

Supporting Information

Thermo-responsive Polymer Micelle with Liquid Crystalline Core

Yoko Mizoue[1], Rintaro Takahashi[2], Kazuo Sakurai[3] and Shin-ichi Yusa[1]

[1] Department of Applied Chemistry, Graduate School of Engineering, University of Hyogo, 2167 Shosha, Himeji, Hyogo 671-2280, Japan; ym85725@gmail.com (Y.M.)

[2] Department of Energy Engineering, Graduate School of Engineering, Nagoya University, Furo-cho, Chikusa-ku, Nagoya 464-8603, Aichi, Japan; r-takahashi@energy.nagoya-u.ac.jp (R.T.)

[3] Department of Chemistry and Biochemistry, University of Kitakyushu, 1-1 Hibikino, Kitakyushu, Fukuoka 808-0135, Japan; sakurai@kitakyu-u.ac.jp (K.S.)

* Correspondence: yusa@eng.u-hyogo.ac.jp (S.Y.)

Table S1. Glass transition (T_g) and melting temperatures (T_m) of PChM and PChM-PNIPAM.

Sample	T_{g1}[a] (°C)	T_{g2}[b] (°C)	T_m (°C)
PChM	51	–	169
PChM-PNIPAM	50	140	171

[a] Glass transition temperature of PChM. [b] Glass transition temperate of PNIPAM.

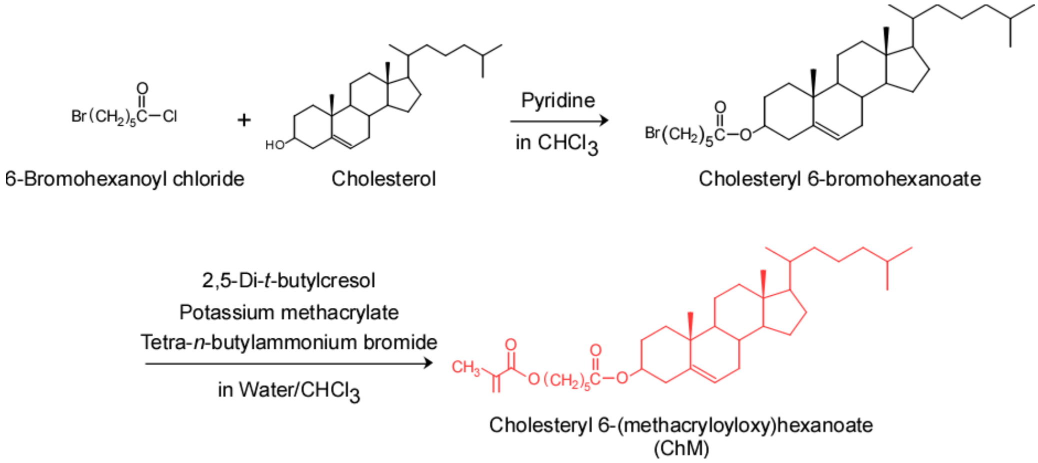

Scheme S1. Synthesis of ChM.

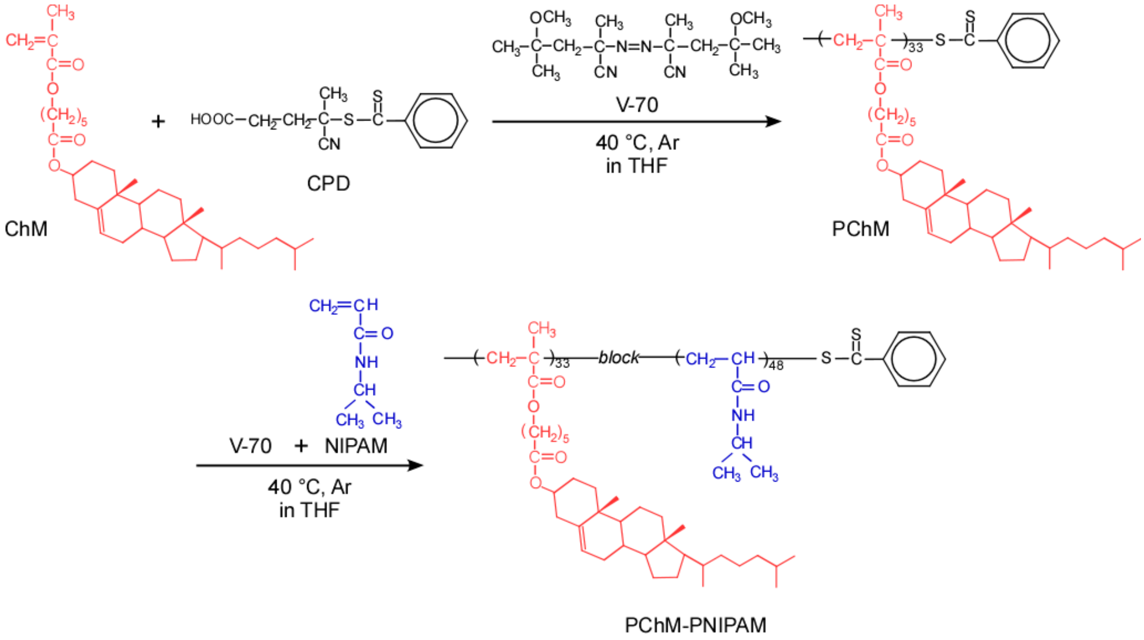

Scheme S2. Synthesis of (a) PChM and (b) PChM-PNIPAM.

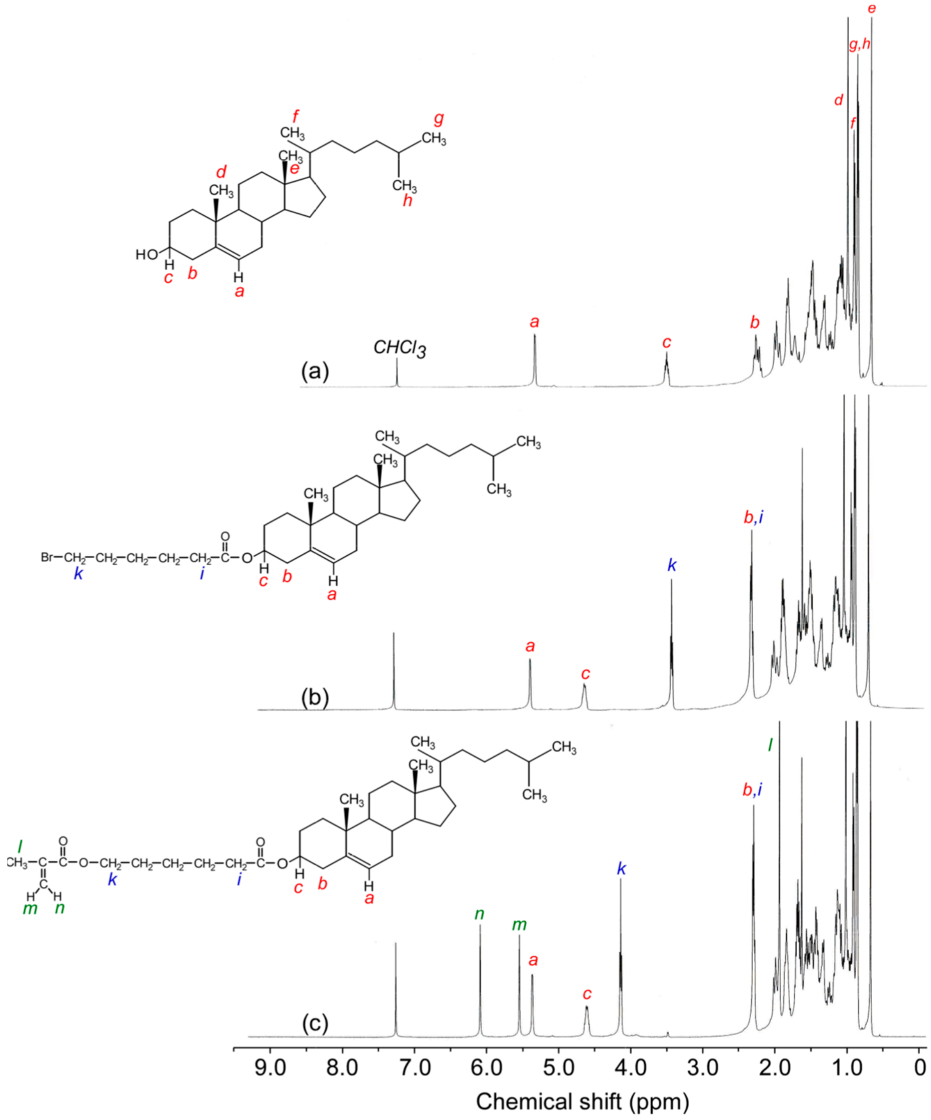

Figure S1. ^{1}H NMR spectra of (a) cholesterol, (b) cholesteryl 6-bromohexanoate, and (c) ChM in $CDCl_3$.

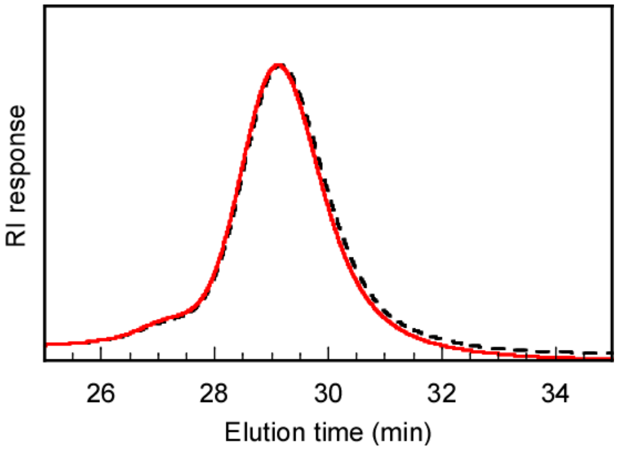

Figure S2. Gel-permeation chromatography (GPC) elution curve for PChM (---) and PChM-PNIPAM (—) using THF as the eluent with a flow rate of 1.0 mL/min at 40 °C.

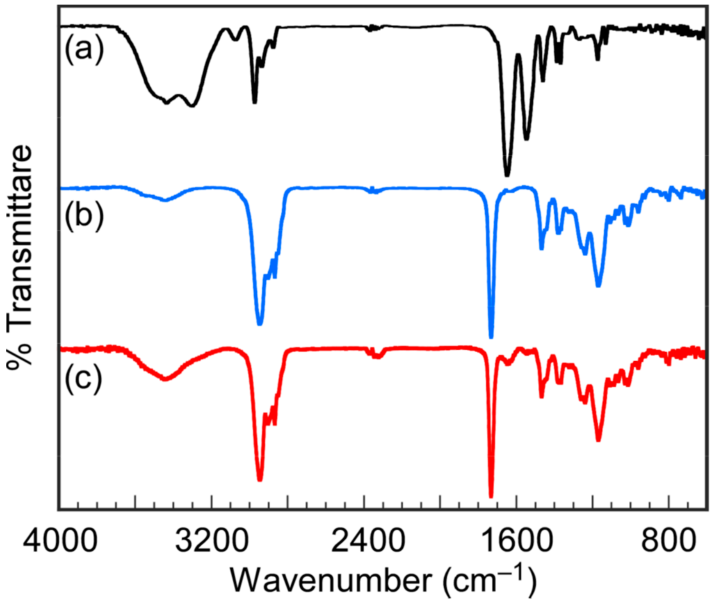

Figure S3. Fourier-transform infrared (FTIR) spectra using KBr pellets for (a) PNIPAM, (b) PChM, and (c) PChM-PNIPAM.

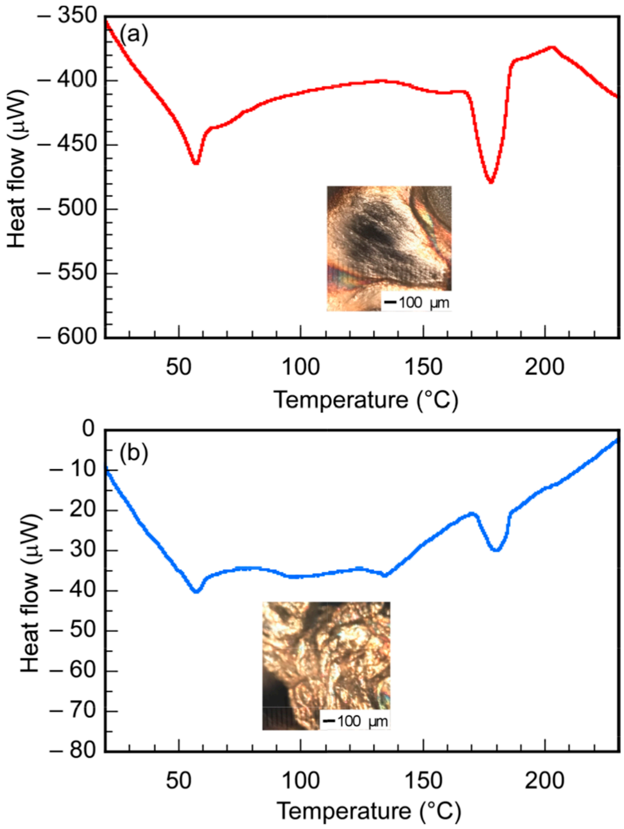

Figure S4. Differential scanning calorimetry (DSC) curves of (a) PChM and (b) PChM-PNIPAM with the second heating processes. The inserts are typical polarizing optical micrographs for (a) PChM at 140 °C and PChM-PNIPAM at 110 °C.

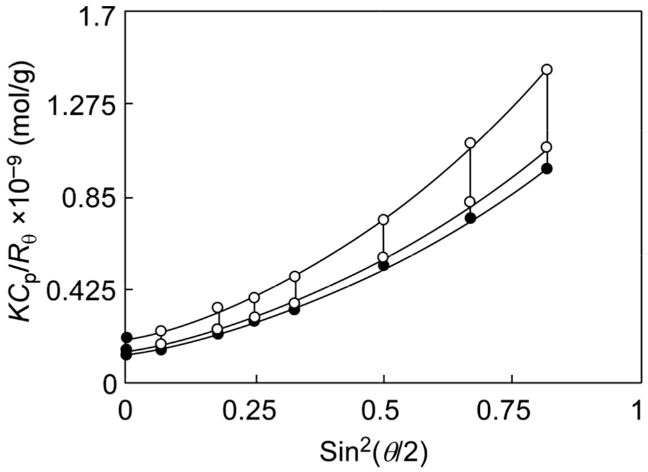

Figure S5. Zimm plot for PChM-PNIPAM in water at 25 °C.

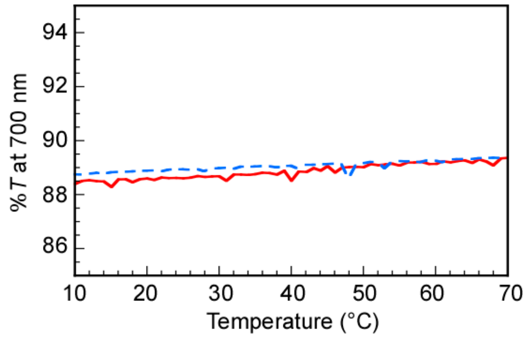

Figure S6. Percentage transmittance (%*T*) for the PChM-PNIPAM aqueous solution with a polymer concentration (C_p) = 0.04 g/L as a function of temperature: heating (—) and cooling processes (---).

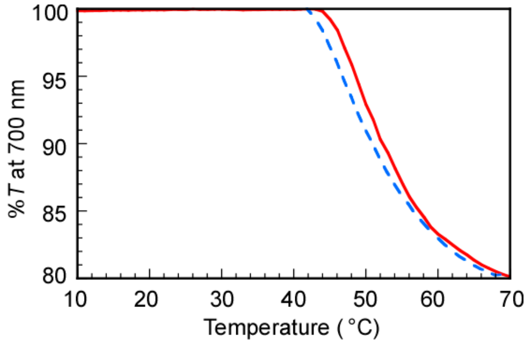

Figure S7. Percentage transmittance (%*T*) of the $PNIPAM_{53}$ with degree of polymerization (DP) = 53 aqueous solution with polymer concentration (C_p) = 0.05 g/L as a function of temperature: heating (—) and cooling processes (---).

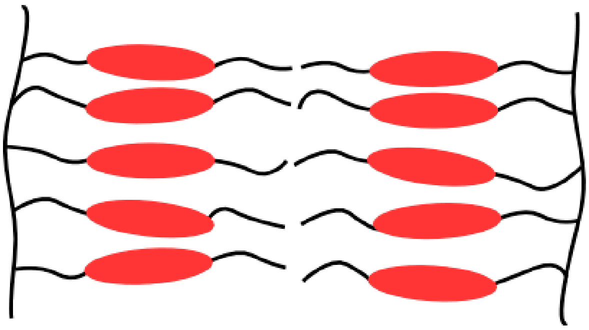

Figure S8. Conceptual illustration of the packing structures of the pendant cholesteryl groups of PChM-PNIPAM in an aqueous solution.
